# 1867. Clinical Presentation and Outcome of Tuberculosis in Patients with Chronic Kidney Disease Stage 4 and 5: A Retrospective Cohort Study from a TB Endemic Country

**DOI:** 10.1093/ofid/ofad500.1695

**Published:** 2023-11-27

**Authors:** Zaheer Udin Babar, Sunil Kumar Dodani, Asma Nasim, Sanjay Kumar Badlani

**Affiliations:** Sindh Institute of Urology and Transplantation, Karachi, Sindh, Pakistan; Sindh Institute of Urology and Transplantation, Karachi, Sindh, Pakistan; Sindh Institute of Urology and Transplantation, Karachi, Sindh, Pakistan; Sindh Institute of Urology and Transplantation, Karachi, Sindh, Pakistan

## Abstract

**Background:**

Patients with chronic kidney disease (CKD) have high risk of developing TB and numerous studies have been done on presentation and outcome of both conditions. However there is a lack of data on regional burden of TB in this group of population. We set out to study on clinical presentation and treatment outcome of TB in patients with moderate to severe renal impairment from a large renal and dialysis center.

Demographics and microbiological characteristics of Tuberculosis in patients with CKD

**Figure:**
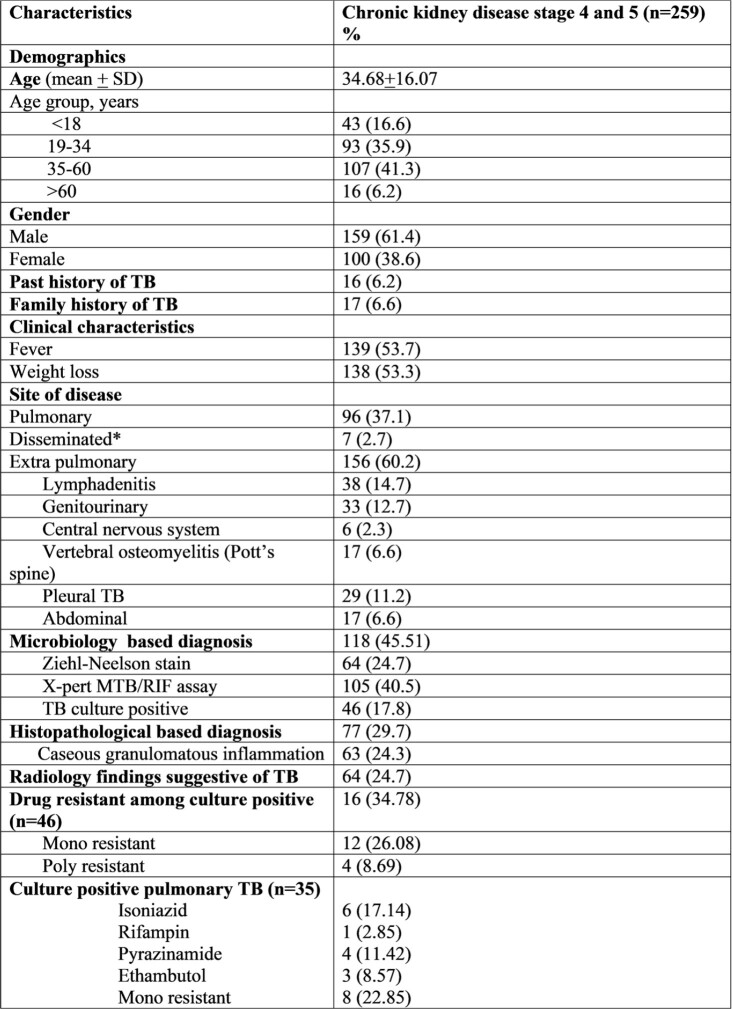

Risk factors of mortality in patients with TB and CKD

**Figure:**
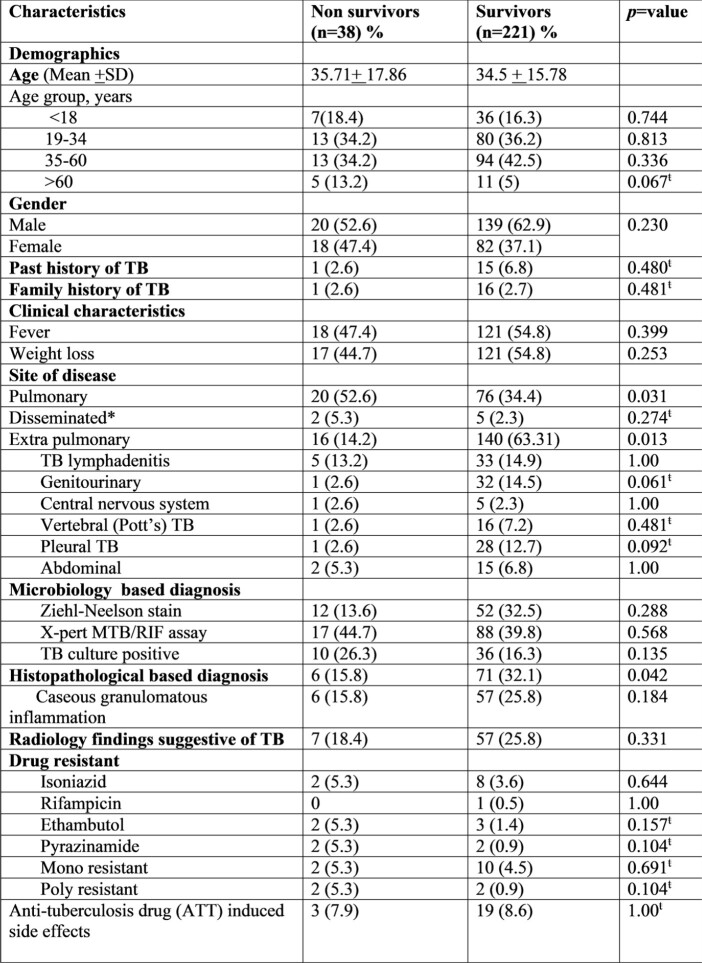

**Methods:**

This is a retrospective cohort study included all patients with CKD stage 4 and 5 and diagnosed active TB from May 2016 till June 2020. Transplant recipients were excluded. TB diagnosis was made as pulmonary tuberculosis (PTB), extra pulmonary TB (EPTB) and disseminated TB (DTB). Primary outcome was measured as treatment successfully completed, lost to follow up, all-cause mortality. Our secondary outcome was to evaluate microbiological characteristics and drug resistant pattern in PTB and EPTB.

Microbiology of mycobacterium TB in CKD patients

**Figure:**
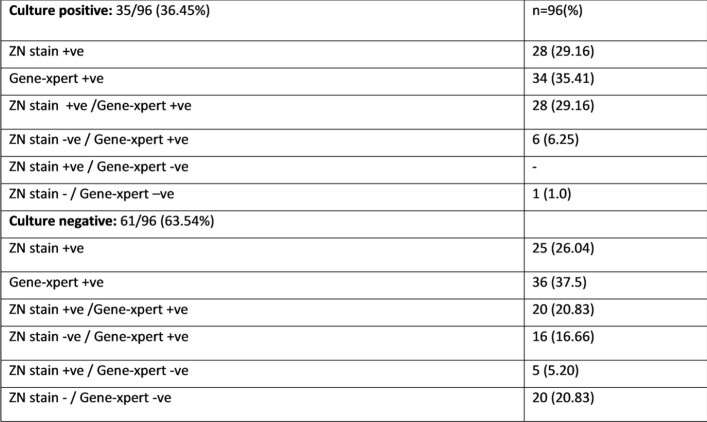

Characteristics of EPTB

**Figure:**
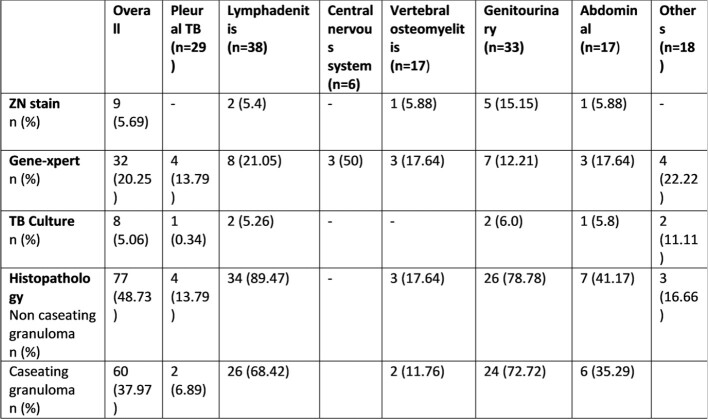

**Results:**

A total of 259 patients with stage 4-5 CKD of all age groups were identified. Mean age of study population was 34.68 +16.7 and a higher proportion of EPTB than PTBTB (61% and 37.1% respectively). Diagnosis of TB in majority of patients 118 (45.51%) were microbiological based and 46 (17.8%) isolates found to have culture positive among those, 16 (34.78%) were resistant to either single (26.08%) or multiple TB drugs (8.69%). Out of 259, 38 (14.7%) patients died during treatment and 12 (4.6%) patients lost to follow up. Our treatment success rate (treatment completed and cured) was 80.7%.

**Conclusion:**

We have made few important observations such as diagnosis EPTB as predominant from in CKD. We have made microbiological based diagnosis in around half of our patients. Drug resistance and mortality was found to be high.

**Disclosures:**

**All Authors**: No reported disclosures

